# Secure Hybrid Deep Learning for MRI-Based Brain Tumor Detection in Smart Medical IoT Systems

**DOI:** 10.3390/diagnostics15050639

**Published:** 2025-03-06

**Authors:** Nermeen Gamal Rezk, Samah Alshathri, Amged Sayed, Ezz El-Din Hemdan, Heba El-Behery

**Affiliations:** 1 Department of Computer and Systems Engineering, Faculty of Engineering, Kafrelsheikh University, Kafrelsheikh 33516, Egypt; nermeen_rezk@eng.kfs.edu.eg (N.G.R.); eng_heba_2010@eng.kfs.edu.eg (H.E.-B.); 2 Department of Information Technology, College of Computer and Information Sciences, Princess Nourah bint Abdulrahman University, P.O. Box 84428, Riyadh 11671, Saudi Arabia; 3 Department of Electrical Energy Engineering, College of Engineering & Technology, Arab Academy for Science Technology & Maritime Transport, Smart Village Campus, Giza 12577, Egypt; 4 Industrial Electronics and Control Engineering Department, Faculty of Electronic Engineering, Menoufia University, Menoufia 32952, Egypt; 5 Department of Computer Science and Engineering, Faculty of Electronic Engineering, Menoufia University, Menoufia 32952, Egypt; ezzeldinhemdan@el-eng.menofia.edu.eg; 6 Structure and Materials Research Lab, Prince Sultan University, Riyadh 12435, Saudi Arabia

**Keywords:** brain tumor detection, deep learning, Medical IoT, secure diagnosis, encrypted MRI images, hybrid models, chaotic and Arnold algorithms

## Abstract

**Background/Objectives**: Brain tumors are among the most aggressive diseases, significantly contributing to human mortality. Typically, the classification of brain tumors is performed through a biopsy, which is often delayed until brain surgery is necessary. An automated image classification technique is crucial for accelerating diagnosis, reducing the need for invasive procedures and minimizing the risk of manual diagnostic errors being made by radiologists. Additionally, the security of sensitive MRI images remains a major concern, with robust encryption methods required to protect patient data from unauthorized access and breaches in Medical Internet of Things (MIoT) systems. **Methods**: This study proposes a secure and automated MRI image classification system that integrates chaotic and Arnold encryption techniques with hybrid deep learning models using VGG16 and a deep neural network (DNN). The methodology ensures MRI image confidentiality while enabling the accurate classification of brain tumors and not compromising performance. **Results:** The proposed system demonstrated a high classification performance under both encryption scenarios. For chaotic encryption, it achieved an accuracy of 93.75%, precision of 94.38%, recall of 93.75%, and an F-score of 93.67%. For Arnold encryption, the model attained an accuracy of 94.1%, precision of 96.9%, recall of 94.1%, and an F-score of 96.6%. These results indicate that encrypted images can still be effectively classified, ensuring both security and diagnostic accuracy. **Conclusions:** The proposed hybrid deep learning approach provides a secure, accurate, and efficient solution for brain tumor detection in MIoT-based healthcare applications. By encrypting MRI images before classification, the system ensures patient data confidentiality while maintaining high diagnostic performance. This approach can empower radiologists and healthcare professionals worldwide, enabling early and secure brain tumor diagnosis without the need for invasive procedures.

## 1. Introduction

Recently, there has been a noticeable rise in interest regarding brain tumor diseases, which severely impact humans and pose life-threatening risks. Brain cancer ranks as the tenth most common primary cause of death among both men and women. According to the International Agency for Research on Cancer, approximately 126,000 individuals globally are diagnosed with brain tumors annually, with their mortality rate exceeding 97,000 [1].

**Figure 1:**
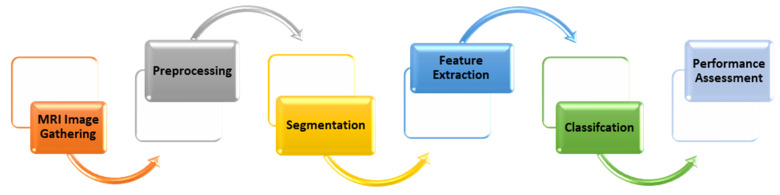
A typical brain tumor detection system.

Early cancer diagnosis is vital for ensuring prompt and effective disease management. Imaging tests, particularly magnetic resonance imaging (MRI), are the primary methods used for diagnosis [2]. However, these tests have certain limitations that can lead to delays in detection and diagnosis. The implementation of computer-aided intelligent systems can assist physicians in making more accurate diagnoses. A typical brain tumor detection system involves many stages, such as image gathering, preprocessing, segmentation, feature extraction, the building of a classification model, and, lastly, the performance assessment, as demonstrated in Figure 1.

**Figure 2:**
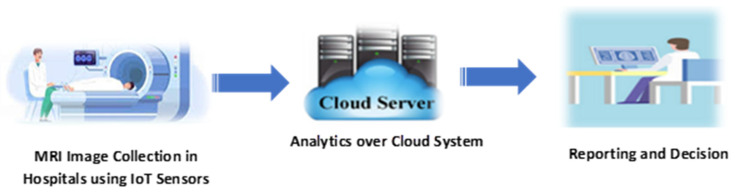
A typical IoT-based Brain Tumor Detection Model.

At the present, the development of artificial intelligence (AI), cloud computing (CC), and the Internet of Things (IoT) has the potential to significantly enhance disease classification and prediction in telemedicine applications [3,4,5,6,7,8]. Machine learning techniques can improve the accuracy and reliability of seizure detection predictions. Recently, the IoT and medical systems have become complementary technologies, offering substantial benefits for various Medical IoT (MIoT) applications. Integrating medical services with IoT devices facilitates real-time monitoring, analysis, and decision-making within complex healthcare systems. For instance, Figure 2 depicts a standard IoT-based Brain Tumor Detection Model: initially, MRI images are gathered in hospitals using IoT sensors; subsequently, these images are stored in the cloud for tumor detection; and finally, doctors can access the data for reporting and decision-making.

Nonetheless, the brain tumor detection-based IoT application framework is powerless and may be effectively assaulted by severe cyber-attacks if a hospital wants to predict the probability of a patient having a tumor through a remote cloud service. In these circumstances, we demand exact predictions and the protection and privacy of the brain data that are transferred. Accordingly, handling encrypted brain data from MIR images effectively has become a critical challenge. The exceedingly challenging security issues in IoT networks have driven us to develop an effective system that surpasses existing solutions. This new system will prioritize classification accuracy and implementation efficiency, and ensure safety and privacy.

Furthermore, brain tumors are complex, with significant variations in size and location, making it difficult to fully understand their nature. MRI analysis requires a professional neurosurgeon, and in developing countries the shortage of skilled doctors and limited knowledge about tumors make generating reports from MRIs challenging and time-consuming. An automated cloud-based system can address this issue effectively. Therefore, this paper introduces a novel approach to encrypted MRI image classification and recognition, combining encryption algorithms such as chaotic and Arnold encryption with hybrid deep learning techniques for secure MIoT applications. The contributions of this study can be summarized as follows:

▪ This paper presents a novel approach to encrypted MRI image classification and recognition by integrating chaotic and Arnold encryption algorithms with hybrid deep learning techniques, significantly enhancing data security and accuracy for secure MIoT applications for brain tumor detection.
▪ The proposed system not only outperforms traditional approaches in terms of accuracy but also addresses cybersecurity challenges in healthcare, contributing to the development of reliable automated systems that can enhance the workflow of neurosurgeons and radiologists, especially in resource-limited settings.
▪ It introduces a hybrid VGG19-DNN model that detects brain tumors in their early stages, accelerating the treatment process and controlling the spread of malignant tissues.
▪ It demonstrates that using a hybrid deep learning classification model, which combines VGG16 with a DNN, results in higher accuracy and better outcomes compared to traditional methods.
▪ The proposed system, which combines VGG16 with a DNN, outperformed other models in both chaotic and Arnold encryption scenarios. With chaotic encryption, it achieved an accuracy of 93.75%, precision of 94.38%, recall of 93.75%, and an F-score of 93.67%. With Arnold encryption, it achieved an accuracy of 94.1%, precision of 96.9%, recall of 94.1%, and an F-score of 96.6%. Additionally, this study employs a new brain tumor classification method that is based on the hybrid VGG19-DNN approach and uses MRI brain images.
▪ It assists radiologists in avoiding errors associated with the manual diagnosis of tumors through MRI images, eliminating the need for invasive measures. Overall, the proposed system can assist and empower doctors worldwide in securely diagnosing brain tumors at an early stage.

This paper is laid out as follows: Section 2 presents prefaces to the Arnold and chaotic Baker map encryption algorithms, while Section 3 explores earlier studies related to the subject of this paper. Section 4 defines the proposed brain tumor detection system, and Section 5 introduces the experimental investigation conducted in this study. Section 6 presents the high-level secure IoT-based brain tumor detection system, while Section 7 offers our conclusions and the future scope of this pioneering work.

## 2. Arnold and Chaotic Encryption Algorithms

**Figure 3:**
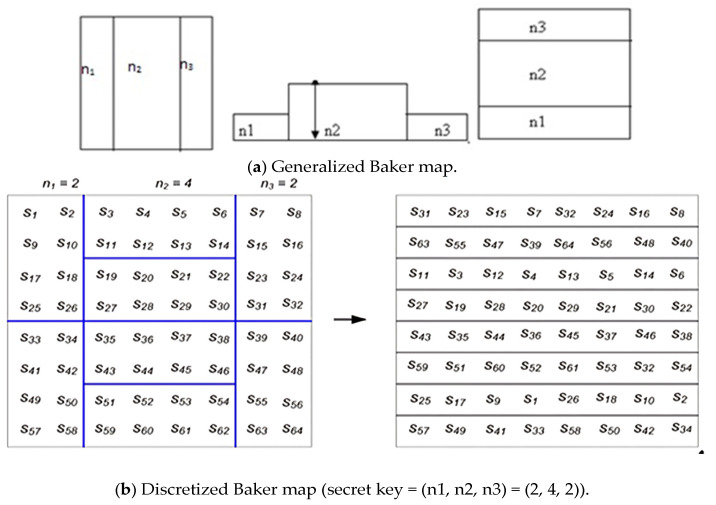
Baker map for an 8 × 8 matrix.

The Arnold algorithm reduces storage and transmission spaces by scrambling pixel positions through iterative number encryption. Meanwhile, the chaotic algorithm, chosen for its robustness in noisy environments compared to diffusion-based methods, encrypts spectrogram images derived from EEG signals in this study. The selection of these permutation algorithms is based on their resilience to attacks in noisy environments [3,4]. The generalized and discretized Baker map, illustrated in Figure 3, is represented by the following principles:

(1) B(x,y)=(2x,y/2)  when 0≤x<1/2,

(2) $$B\left(x,y\right)=\left(2x-1,\frac{y}{2}+\frac{1}{2}\right)\;\;when\;\;\;\;\frac{1}{2}\leq x\leq 1$$

The discretized map can be illustrated using the subsequent formulas:

(3) $$B_{(n_{1},\text{.....},n_{k)}}(r,s)=\left[\frac{N}{n_{i}}\left(r-N_{i}\right)+s\mathrm{mod}\left(\frac{N}{n_{i}}\right),\frac{n_{i}}{N}\left(s-s\mathrm{mod}\left(\frac{N}{n_{i}}\right)\right)+N_{i}\right]$$

where B_(n1,n2,……,nk)_ and (r,s) are the new indices of the data item at (r,s), $N_{i}\leq r<N_{i}+n_{i}$, 0≤s<N, and N_i_ = n_1_ + n_2_ + …… + n_i_.

## 3. VGG19

**Figure 4:**
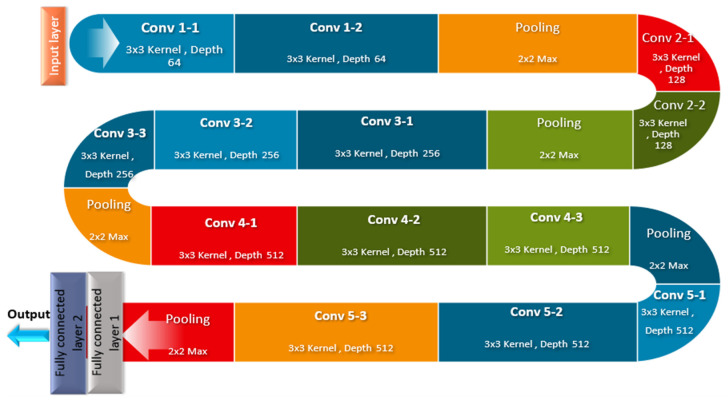
Basic VGG 16 neural network.

One of the most common deep convolutional neural networks used in image recognition is the Visual Geometry Group (VGG 16). The VGG16 has been proven to be a powerful technique for feature extraction in medical imaging applications [5,6]. It has been successfully utilized in various medical imaging tasks, including the detection and classification of diseases. VGG16’s robust architecture enables it to capture intricate details in medical images, making it a valuable tool for improving diagnostic accuracy and patient outcomes. As shown in Figure 4, this model consists of 16 layers of neurons with weights, where each layer acts incrementally on the information in the image and improves the prediction’s accuracy.

VGG16 utilizes convolution layers with a 3 × 3 filter and a stride of 1 that are in the same padding and maxpool layer as a 2 × 2 filter with a stride of 2, without the need for a large number of hyper-parameters. Throughout the whole architecture, the convolution and max pool layer arrangements are maintained. Ultimately, the network consists of two fully connected layers with a softmax as the output. The main advantage of this model is that it is available online and free to download and use in any system or application.

## 4. Previous Studies

The advances in artificial intelligence techniques, especially machine learning and deep learning, play a critical role in improving medical image analysis and the detection of cancers. Many researchers have investigated various techniques to improve their classification, robustness, and security in medical imaging applications. These developments have contributed to earlier and more precise diagnoses while ensuring the protection of sensitive medical data.

### 4.1. Machine Learning for Cancer Detection

In recent years, the evolution of smart healthcare systems has played a crucial role in improving cancer detection, particularly through machine learning techniques. This progress has driven the significant research efforts being made toward leveraging advanced learning models for the detection and classification of various types of cancer, including brain tumors. These studies focus not only on improving diagnostic accuracy but also on optimizing treatment planning and patient outcomes. By utilizing machine learning and image analysis algorithms, researchers are developing innovative approaches to enhance the efficiency and reliability of cancer detection. These advancements hold great potential for transforming medical diagnostics, enabling more accurate, automated, and personalized cancer detection systems. Paper [6] presents the new Deep Transfer for Bone Cancer Diagnosis (DTBV) system. The system employs transfer learning to enhance the accuracy of bone cancer detection by extracting critical features from pre-processed X-ray images, selecting the most relevant ones using mutual information statistics, and classifying them into malignant or benign categories. The authors of [7] introduced a deep learning model designed for multi-class and binary cancer classification using histopathological images of breast, colon, and lung cancers. By significantly reducing diagnostic errors and human biases, the machine learning model led to a substantial advancement in computer-aided cancer diagnosis, improving efficiency, accuracy, and the automation of clinical workflows.

### 4.2. Deep Learning and Ensemble Learning

The paper [8] introduced a hybrid deep learning framework for early Alzheimer’s disease diagnosis with an adaptive weight selection process optimized using the Cuckoo Search algorithm. The framework enhances classification accuracy, particularly for early-stage detection. The authors of [9] introduced a new medical image denoising model, which used an end-to-end learning framework. The model employed a deep wider residual block to capture long-distance pixel dependencies and employed the multi-head-attention-guided reconstruction of images for the effective denoising of medical images. In contrast, the authors of [10] described a mesh network built to preserve features during retinal vascular semantic segmentation. The method aimed to assist in the evaluation of ophthalmic diseases by retaining important structural information within retinal images. Paper [11] sheds light on the latest deep learning models for brain tumor segmentation through multi-modal MRI data. The methods are classified into CNNs, vision transformer-based models, and hybrid architectures, with a detailed comparison of their performance and discussion of open research challenges.

In [12], a new abnormal brain disease classification approach that uses transfer learning was proposed, while another study [13] presented an ensemble learning-based method for brain image classification as either tumor or non-tumor. The approach combined preprocessing, segmentation, and feature extraction techniques to enhance classification accuracy. Similarly, [14] introduced an algorithm that utilizes MRI images for brain tumor segmentation, feature extraction, and classification, and includes a signal-to-noise ratio method to mitigate extraneous noise.

### 4.3. Secure Medical Imaging

Several studies have explored different classification techniques for MRI brain images that can improve security and diagnostic accuracy. Study [15] focused on classifying MRI brain images into normal and abnormal tissue categories using GLCM, LBP, HOG, and a k-NN classifier. Similarly, ref. [16] presented an approach to brain tumor recognition using MRI images, while [17] developed a method for brain tumor detection and feature extraction from MRI images. In [18], a novel methodology was introduced that combines CNN architecture with neutrosophic expert maximum-fuzzy (NS-CNN) for brain tumor classification. These methods emphasize the importance of both accuracy and security in medical imaging applications, ensuring that deep learning models remain robust while preserving image integrity.

A comparison table (Table 1) summarizes the key contributions of each study mentioned, outlining the main methods, areas of focus, and techniques applied in each referenced work.

Previous research has not focused on the classification of encrypted MRI images to safeguard sensitive data during transmission over insecure communication channels. Hence, developing an automated and secure system for effective brain tumor detection within Medical Internet of Things (MIoT) environments is crucial. This approach aims to assist radiologists in diagnosing tumors non-invasively, using MRI images. However, ensuring the security of these sensitive MRI images is paramount to prevent unauthorized access and potential breaches across vulnerable networks. Therefore, an efficient encryption method is urgently needed to maintain confidentiality during classification and prediction processes. This study proposes a novel methodology for encrypted MRI image classification and recognition, integrating chaotic and Arnold encryption algorithms with hybrid deep learning techniques tailored for secure MIoT applications. While encryption does introduce an additional processing time, these techniques are lightweight compared to conventional cryptographic methods and optimized for real-time implementation. Our proposed encryption approach is designed to provide robust security while maintaining computational efficiency. Thus, this encryption method is adequate in clinical workflows and in emergency scenarios.

## 5. Proposed System

**Figure 5:**
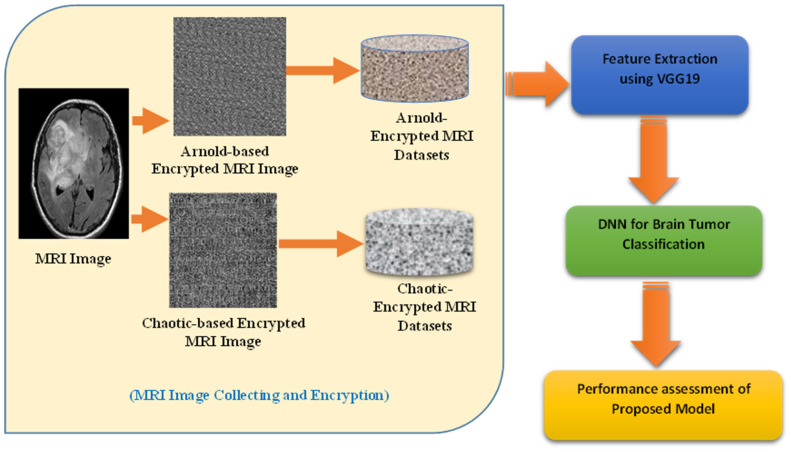
Proposed hybrid VGG19-DNN system for tumor detection.

This section presents the proposed hybrid VGG19-DNN system for brain tumor detection and classification. The primary goal of this work is to ensure the secure detection of brain tumors from MRI scans. The proposed approach incorporates chaotic and Arnold encryption techniques to protect MRI images over Medical IoT networks. Following encryption, a hybrid deep learning model utilizing VGG19 is employed to detect brain tumors from the encrypted images. The key stages of this system are illustrated in Figure 5. These stages are (1) MRI Image Collection and Encryption (MICE), (2) Feature Extraction from MIR Images (FEMI) using VGG19, (3) DDN-based Brain Tumor Detection (DBTD), and (4) a Brain Tumor Detection Assessment (BTDA). The explanations of these stages are as follows:

▪ Stage 1: MRI Image Collection and Encryption (MICE)
  - MRI images are collected. Then, to ensure data security over Medical IoT networks, the images undergo chaotic and Arnold transforms before further processing.
  - Chaotic Encryption: A chaotic map-based transformation scrambles pixel positions non-linearly, increasing randomness and reducing redundancy.
  - Arnold Transform: This is a periodic transformation that reorders pixel positions, preventing direct visual interpretation.
  - Impact on Feature Distribution: These transformations disrupt spatial correlations while preserving intrinsic patterns, ensuring that deep learning models can still extract meaningful representations.
▪ Stage 2: Feature Extraction from MRI Images (FEMI) using VGG19
  - The encrypted MRI images are resized to 224 × 224 pixels and normalized to align with the input requirements of the deep learning model.
  - A pre-trained VGG19 model is employed to extract deep feature representations from the encrypted images.
  - Unlike conventional models that operate on raw images, VGG19 processes encrypted images, demonstrating the resilience of deep networks in handling transformed inputs.
  - Feature Representation: The final fully connected (FC) layer of VGG19 is removed, and the extracted feature maps serve as input for the classification stage.
▪ Stage 3: DNN-based Brain Tumor Detection (DBTD)
  - The extracted deep features are fed into a custom deep neural network (DNN) for classification.
  - Difference from CNN-based classifiers:
    ○ Unlike a conventional CNN-based classifier, the DNN leverages extracted features from VGG19 to focus on high-level feature learning.
    ○ The DNN consists of multiple fully connected layers with ReLU activation followed by a Softmax layer to classify the images into tumor and non-tumor categories.
    ○ This separation of feature extraction (VGG19) and classification (DNN) enhances computational efficiency and prevents overfitting, particularly with encrypted inputs.
▪ Stage 4: Brain Tumor Detection Assessment (BTDA)
  - The performance of the proposed hybrid VGG19-DNN model is evaluated using various experimental scenarios.
  - The key evaluation metrics used include accuracy, precision, recall, F1-score, and AUC-ROC curves.
  - A comparative analysis with traditional CNN classifiers is performed to highlight the robustness of using encrypted images in deep learning-based tumor detection.

## 6. Experimental Study

This section outlines the experimental environment and the brain tumor dataset used in this study. It also presents the results obtained with the proposed system, followed by a thorough analysis and discussion of these findings to evaluate the system’s effectiveness.

A. Brain Tumor Dataset

**Figure 6:**
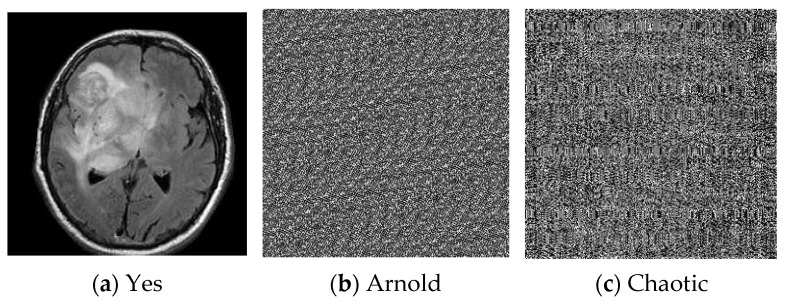

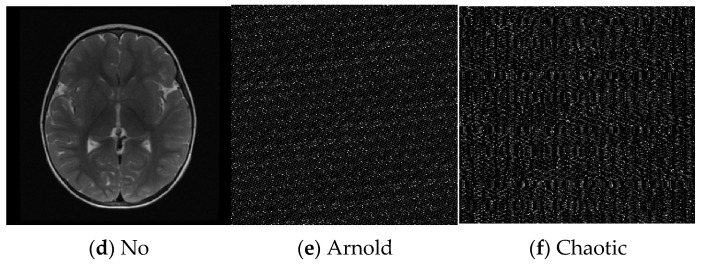
Encrypted MRI images for normal and tumor cases.

The experiments were conducted on a brain tumor dataset [19] which includes 251 images: 97 normal images and 154 tumor images. Figure 6 illustrates examples of the MRI images used in this study, showcasing both normal (no) and tumor (yes) conditions. These images have been encrypted using the Arnold and chaotic encryption algorithms to ensure data security during processing.

B. Evaluation Metrics

The performance evaluation of the proposed hybrid approach in terms of the brain tumor classification and prediction objective can be carried out using performance metrics like accuracy, precision, recall, and F1-score, which are calculated as per Equations (4)–(7), respectively, using the parameters in the confusion matrix presented in Table 2 [20,21,22].

(4) $$Accuray=\frac{TP+TN}{TP+FP+FN+TN}$$

(5) $$\;\;\;\;\;\;Precision=\frac{TP}{TP+FP\;\;\;\;\;\;\;\;\;\;}$$

(6) $$Recall=\frac{TP}{TP+FN}$$

(7) $$F1\;Score=\frac{2*(Recall*Precision)}{Recall+Precision}$$

C. Analysis of the Results

In this section, we discuss the classification results obtained using the proposed approach and the chaotic and Arnold Encryption algorithms. Table 3 below summarizes the performance of various algorithms in detecting brain tumors when using chaotic encryption on MRI images. From this table, the evaluation of various deep learning models’ performance on brain tumor images encrypted using chaotic encryption methods reveals significant differences. VGG16 accomplished an accuracy of 75%, improving to 81.25% with VGG19, indicating better feature extraction capabilities in the deeper architecture. Xception proved a lower accuracy, at 70%, while DenseNet showed an 80% accuracy with better recall. ResNetV3 had a notably low accuracy of 66%, suggesting it had difficulty in learning encrypted image features, whereas MobileNet, with an 80% accuracy, exhibited a balanced performance across precision, recall, and F-score. Inception had the lowest performance, with a 50% accuracy. The Autoencoder + DNN model performed better, with an accuracy of 87.5% and strong precision and recall values. The proposed hybrid VGG16 + DNN system outperformed all other models, achieving the highest accuracy, 93.75%, and a superior precision, recall, and F-score of around 94%, demonstrating the robustness of combining VGG16’s feature extraction with a DNN’s classification strength.

On the other hand, Table 4 below summarizes the performance of various algorithms in detecting brain tumors when using Arnold encryption on MRI images. From this table, the assessment of the performance of different deep learning models on brain tumor images encrypted using Arnold encryption techniques shows significant differences. VGG16 achieved an accuracy of 71.9%, while VGG19 increased this to 77.19%, implying a better performance with the deeper architecture. Xception demonstrated a slightly higher accuracy at 77.7%, with balanced precision and recall. DenseNet showed an accuracy of 75.4%, performing well but with some inconsistency in its precision and recall. ResNetV3 had a lower accuracy of 66%, suggesting it had difficulty in processing the encrypted images. MobileNet, with an accuracy of 80%, exhibited a good overall performance across the metrics. Inception showed a lower performance, with a 61.1% accuracy.

The Autoencoder + DNN model performed significantly better, achieving an accuracy of 85% with strong precision and recall values. The proposed hybrid VGG16 + DNN system outperformed all other models, achieving the highest accuracy, 94.1%, and a superior precision and F-score of around 96.9%, revealing its robustness in handling encrypted brain tumor images.

**Figure 7:**
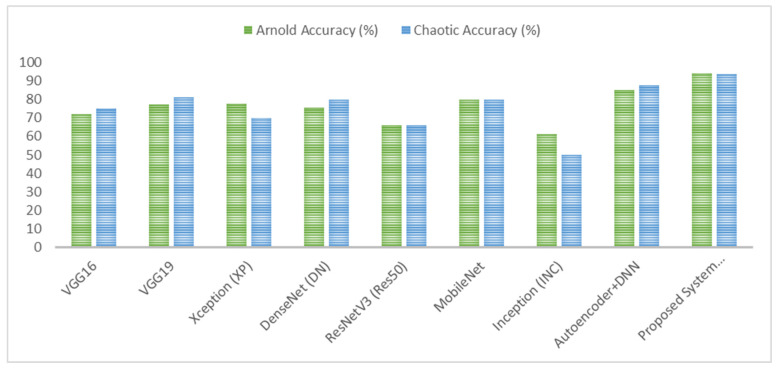
Results of the proposed system’s accuracy when using Arnold and chaotic encryption algorithms.

**Figure 8:**
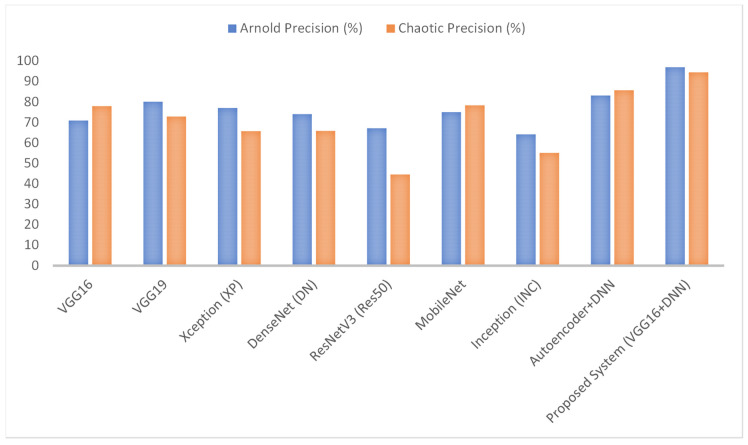
Results of the proposed system’s precision when using Arnold and chaotic encryption algorithms.

**Figure 9:**
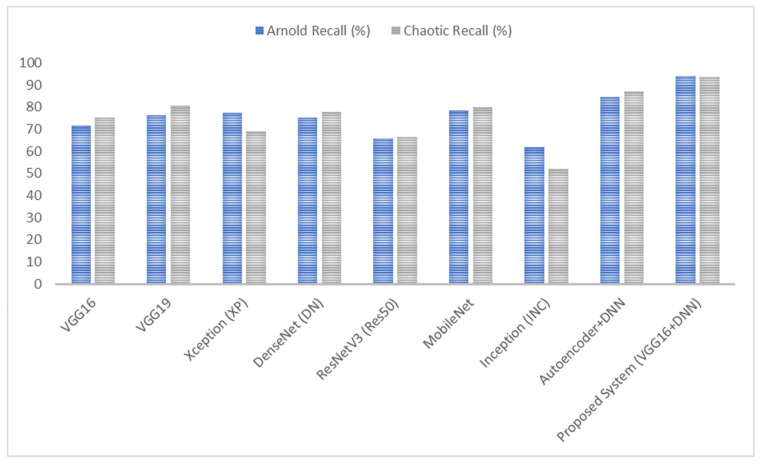
Results of the proposed system’s recall when using Arnold and chaotic encryption algorithms.

**Figure 10:**
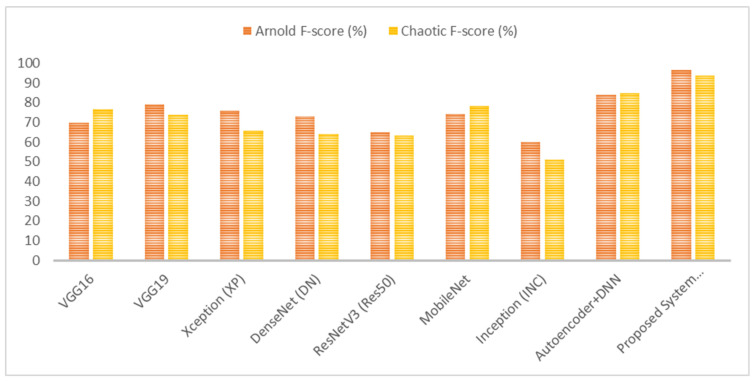
Results of the proposed system’s F1-score when using Arnold and chaotic encryption algorithms.

As shown in Figure 7, Figure 8, Figure 9 and Figure 10, the proposed system (VGG16 + DNN) achieves the highest scores in all metrics with both chaotic and Arnold encryptions, with accuracies of 93.75% and 94.1% and F-scores of 93.67% and 96.6%. Autoencoder + DNN achieves the next strongest performance, particularly with Chaotic encryption (87.5% accuracy and 85.0% F-score). Inception and ResNetV3 have the lowest scores in all metrics across both encryption methods.

In summary, the proposed system, combining VGG16 with a DNN, outperformed other models with chaotic encryption, achieving the highest accuracy of 93.75%, a precision of 94.38%, recall of 93.75%, and F-score of 93.67%. On the other hand, the proposed system, combining VGG16 with a DNN, outperformed other models with Arnold encryption, achieving the highest accuracy of 94.1%, a precision of 96.9%, recall of 94.1%, and F-score of 96.6%. The superior performance of Arnold encryption is due to its pixel-scrambling methods which, compared with chaotic transformations, maintain local structural patterns more efficiently. Also, chaotic encryption creates high randomness, while Arnold transformations maintain spatial relationships to extract meaningful features by deep learning and meet security requirements.

Additionally, the exceptional performance of the proposed system highlights the importance of integrating advanced deep learning techniques for secure medical image analysis. Its high accuracy and consistent performance metrics underscore its potential for real-world applications, providing radiologists with a reliable tool for early and accurate tumor detection without invasive procedures. The use of Arnold and chaotic encryption techniques ensures that MRI images are protected against unauthorized access during transmission and storage. The system’s effectiveness in handling encrypted images underscores its applicability in secure Medical Internet of Things (MIoT) environments. Future research could explore additional encryption methods and advanced neural network architectures to further enhance performance and security. In conclusion, the hybrid VGG16-DNN model presents a promising approach to secure and efficient brain tumor detection, paving the way for improved diagnostic tools in medical imaging.

## 7. High-Level Secure IoT-Based Brain Tumor Detection System

**Figure 11:**
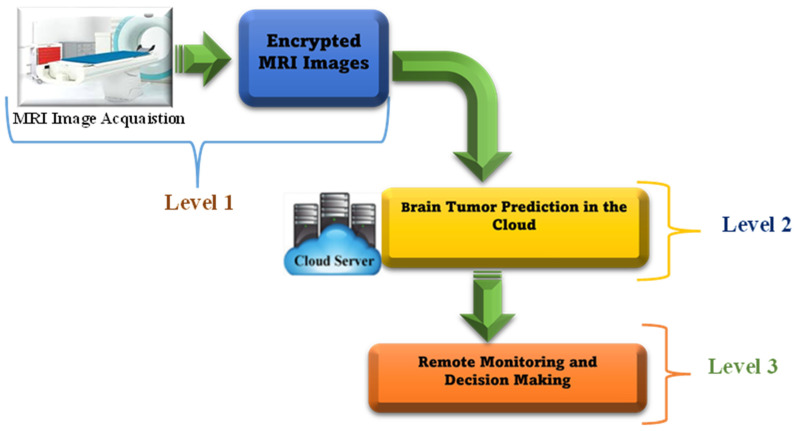
Proposed remote monitoring framework for initial brain tumor detection.

Realistically serving as a practical smart medical IoT system, this proposed system heightens the efficacy of healthcare. This system has three key levels, each with defined tasks and methods, that work together to accomplish the scheme’s purposes, as clarified in Figure 11:

1. Level 1: Gathering MRI images using smart medical IoT devices in hospitals and then immediately encrypting them.
2. Level 2: Submission of the gathered encrypted MRI images to cloud servers for prediction and classification purposes. This level uploads patient data from connected smart IoT devices, making them available for medical investigations.
3. Level 3: Doctors and medical workers use a cloud-based dashboard tracking system to monitor patient brain tumor indicators. The endorsed system creates reports through cloud-based hybrid model predictions, allowing staff to review and determine the appropriate procedures following brain tumor detection.

A comparison with previous methods was performed and included as the performance evaluation shown in Table 5. Table 5 presents a comparative analysis of the performance metrics of various models for MRI image classification. Existing methods include GLCM combined with K-means and k-NN, which achieved an accuracy of 85.0%, and Alex-Net CNN, which achieved an accuracy of 91.2%. The NS-CNN combined with SVM performed best among the existing works, achieving an accuracy of 95.6%. In contrast, the proposed system, which utilizes multiple deep learning models (VGG19, Xception, DenseNet, ResNetV3, MobileNet, Inception, and Autoencoder + DNN), demonstrated a superior performance, with an accuracy of 93.75% when using Arnold encryption and 94.1% when using chaotic encryption. Additionally, the proposed system excelled in precision and recall, particularly with Arnold encryption, achieving a 96.9% precision and 94.1% recall.

The limitations of this study are the following:

- Latency Concerns: The encryption and cloud-based processing pipeline may introduce latency, raising concerns about its feasibility in time-sensitive emergency scenarios.
- Impact of Diagnostic Speed: The effect of encryption on the speed of diagnosis, particularly in settings with limited computational resources, may hinder its real-time application.
- Performance on Diverse Datasets: While the system has demonstrated high accuracy, its effectiveness in handling diverse and complex real-world datasets requires further investigation.
- False Positives/Negatives: The potential for false positives or negatives under encrypted conditions requires additional scrutiny of the system to ensure its reliability.
- Integration Challenges: The ease of the proposed system’s integration into existing clinical workflows, especially in resource-limited environments, should be evaluated to ascertain its practical utility.

## 8. Conclusions and Future Scope

In recent years, brain tumor detection via IoT frameworks has been vulnerable to severe cyber-attacks, necessitating secure and precise remote cloud predictions. The complexity of tumors and the shortage of skilled neurosurgeons, especially in developing countries, highlight the need for automated systems. Therefore, this paper presents a novel approach that uses encrypted MRI image classification, with chaotic and Arnold encryption algorithms and hybrid deep learning techniques, for secure MIoT applications. From the obtained results, the proposed system, combining VGG16 with a DNN, demonstrated a superior performance compared to other models in both chaotic and Arnold encryption scenarios. Achieving an accuracy of 93.75% with chaotic encryption and 94.1% with Arnold encryption, it outperformed alternative approaches significantly. Other models exhibited varying performances: VGG19 achieved an accuracy of 81.25%, Xception 77.7%, DenseNet 75.4%, ResNetV3 66.0%, MobileNet 80.0%, Inception 61.1%, and Autoencoder + DNN 85.0%. These results highlight the effectiveness of the proposed approach in accurately detecting brain tumors from encrypted MRI images while surpassing the performance of other state-of-the-art models.

Potential future trends and open points for this research can be summarized as follows:

- Integration of Advanced Encryption Methods: Researchers could explore and implement more sophisticated encryption techniques to further enhance data security in MRI image classification systems.
- Deployment of Edge Computing: Researchers could utilize edge computing to facilitate real-time processing, reducing latency and improving the efficiency of the proposed system in clinical settings.
- Validation Through Clinical Trials: Extensive clinical trials could be conducted to validate the performance and reliability of this system in real-world medical environments.
- Enhancement of AI Explainability: Research could focus on improving AI explainability to provide insights into its decision-making processes for clinicians and patients.
- Addressing Global Accessibility Challenges: Strategies could be developed to ensure that advanced medical technologies are accessible to diverse populations, particularly in resource-limited settings.
- Ensuring Regulatory Compliance: Researchers should work towards compliance with healthcare regulations and guidelines to uphold ethical standards on the use of patient data, particularly in encrypted MRI image classification systems for brain tumor detection.

## Figures and Tables

**Table 1:** Summaries of previous studies.

Study	Methodology	Focus	Techniques
[12]	Transfer Learning	Abnormal Brain Disease Classification	Transfer learning approach for disease classification
[13]	Ensemble Learning	Brain Image Classification (Tumor/Non-Tumor)	Pre-processing, segmentation, and feature extraction
[14]	Algorithm for MRI Segmentation	Brain Tumor Segmentation and Classification	Signal-to-noise ratio for noise reduction, segmentation, and feature extraction
[15]	MRI Image Classification	Normal/Abnormal Tissue Classification	GLCM, LBP, HOG, and k-NN classifier
[16]	MRI Image-Based Recognition	Brain Tumor Recognition	MRI-based detection methods
[17]	Feature Extraction and Tumor Detection	Brain Tumor Detection	MRI-based tumor detection and feature extraction
[18]	NS-CNN (CNN with Neutrosophic Expert System)	Brain Tumor Classification	CNN architecture, neutrosophic expert maximum-fuzzy (NS-CNN)

**Table 2:** The parameters of confusion matrix used for brain tumor detection.

	Predicted Yes	Predicted No
Actual Yes	TP	FP
Actual No	FN	TN

**Table 3:** Model performance on brain tumor images encrypted using chaotic encryption.

Algorithm	Accuracy (%)	Precision (%)	Recall (%)	F-Score (%)
VGG16	75	77.9	75.1	76.5
VGG19	81.25	72.8	80.6	73.8
Xception (XP)	70	65.7	69	65.7
DenseNet (DN)	80	65.8	77.9	64
ResNetV3 (Res50)	66	44.5	66.6	63.3
MobileNet	80	78.3	79.8	78.2
Inception (INC)	50	55	52.1	51.2
Autoencoder + DNN	87.5	85.7	87.2	85
Proposed System (VGG16 + DNN)	93.75	94.38	93.75	93.67

**Table 4:** Model performance on brain tumor images encrypted using Arnold encryption.

Algorithm	Accuracy (%)	Precision (%)	Recall (%)	F-Score (%)
VGG16	71.9	70.8	71.7	70.0
VGG19	77.19	80.0	76.5	79.1
Xception (XP)	77.7	77.0	77.5	76.0
DenseNet (DN)	75.4	74.0	75.2	73.0
ResNetV3 (Res50)	66.0	67.0	65.8	65.0
MobileNet	80.0	74.9	78.4	74.2
Inception (INC)	61.1	64.1	62.0	60.2
Autoencoder + DNN	85.0	83.0	84.5	84.0
Proposed System (VGG16 + DNN)	94.1	96.9	94.1	96.6

**Table 5:** A comparative study between the proposed system and existing works, based on performance metrics.

Ref.	Model	Dataset	Accuracy	Precision	Recall
[23]	GLCM + K-mean + k-NN	MRI	85.0%	-	-
[24]	Alex-Net CNN	MRI	91.2%	-	-
[25]	NS-CNN +SVM	MRI	95.6%		
Proposed System	Deep Learning Models (VGG19, Xception, DenseNet, ResNetV3, MobileNet, Inception and, Autoencoder + DNN)	Encrypted MRI using Arnold	93.75%	96.9%	94.1%
		Encrypted MRI using chaotic	94.1%	94.38%	93.75%

## Data Availability

The original contributions presented in this study are included in the article; further inquiries can be directed to the corresponding author.

## References

[1] Ferlay J., Soerjomataram I., Dikshit R., Eser S., Mathers C., Rebelo M., Parkin D.M., Forman D., Bray F. (2015). Cancer incidence and mortality worldwide: Sources, methods and major patterns in GLOBOCAN 2012. Int. J. Cancer.

[2] Anagun Y. (2023). Smart brain tumor diagnosis system utilizing deep convolutional neural networks. Multimed. Tools Appl..

[3] Khan M.U., Kamran M.A., Khan W.R., Ibrahim M.M., Ali M.U., Lee S.W. (2024). Error Mitigation in the NISQ Era: Applying Measurement Error Mitigation Techniques to Enhance Quantum Circuit Performance. Mathematics.

[4] Ali M.U., Kim K.S., Khalid M., Farrash M., Zafar A., Lee S.W. (2024). Enhancing Alzheimer’s disease diagnosis and staging: A multistage CNN framework using MRI. Front. Psychiatry.

[5] Sharma S., Guleria K., Tiwari S., Kumar S. (2022). A deep learning based convolutional neural network model with VGG16 feature extractor for the detection of Alzheimer Disease using MRI scans. Meas. Sens..

[6] Suganeshwari G., Balakumar R., Karuppanan K., Prathiba S.B., Anbalagan S., Raja G. (2023). DTBV: A Deep Transfer-Based Bone Cancer Diagnosis System Using VGG16 Feature Extraction. Diagnostics.

[7] Ejiyi C.J., Qin Z., Agbesi V.K., Yi D., Atwereboannah A.A., Chikwendu I.A., Bamisile O.F., Kissanga G.M.B., Bamisile O.O. (2025). Advancing cancer diagnosis and prognostication through deep learning mastery in breast, colon, and lung histopathology with ResoMergeNet. Comput. Biol. Med..

[8] Gasmi K., Alyami A., Hamid O., Altaieb M.O., Shahin O.R., Ben Ammar L., Chouaib H., Shehab A. (2024). Optimized Hybrid Deep Learning Framework for Early Detection of Alzheimer’s Disease Using Adaptive Weight Selection. Diagnostics.

[9] Naqvi R.A., Haider A., Kim H.S., Jeong D., Lee S.-W. (2024). Transformative Noise Reduction: Leveraging a Transformer-Based Deep Network for Medical Image Denoising. Mathematics.

[10] Imran S.M.A., Saleem M.W., Hameed M.T., Hussain A., Naqvi R.A., Lee S.W. (2023). Feature preserving mesh network for semantic segmentation of retinal vasculature to support ophthalmic disease analysis. Front. Med..

[11] Abidin Z.U., Naqvi R.A., Haider A., Kim H.S., Jeong D., Lee S.W. (2024). Recent deep learning-based brain tumor segmentation models using multi-modality magnetic resonance imaging: A prospective survey. Front. Bioeng. Biotechnol..

[12] Kaur T., Gandhi T.K. Automated brain image classification based on VGG-16 and transfer learning. Proceedings of the 2019 International Conference on Information Technology (ICIT).

[13] Kumar P., VijayKumar B. (2019). Brain tumor MRI segmentation and classification using ensemble classifier. Int. J. Recent Technol. Eng. IJRTE.

[14] Bahadure N.B., Ray A.K., Thethi H.P. (2017). Image analysis for MRI based brain tumor detection and feature extraction using biologically inspired BWT and SVM. Int. J. Biomed. Imaging.

[15] Khalil M., Ayad H., Adib A. (2018). Performance evaluation of feature extraction techniques in MR-Brain image classification system. Procedia Comput. Sci..

[16] Leo M.J. (2019). MRI brain image segmentation and detection using K-NN classification. J. Phys. Conf. Ser..

[17] Kabir M.A. (2020). Automatic brain tumor detection and feature extraction from mriimage. GSJ.

[18] Özyurt F., Sert E., Avci E., Dogantekin E. (2019). Brain tumor detection based on Convolutional Neural Network with neutrosophic expert maximum fuzzy sure entropy. Measurement.

[19] https://www.kaggle.com/datasets/navoneel/brain-mri-images-for-brain-tumor-detection.

[20] Rezk N.G., Alshathri S., Sayed A., El-Din Hemdan E., El-Behery H. (2024). XAI-Augmented Voting Ensemble Models for Heart Disease Prediction: A SHAP and LIME-Based Approach. Bioengineering.

[21] Torkey H., Hashish S., Souissi S., Hemdan E.E.-D., Sayed A. (2025). Seizure Detection in Medical IoT: Hybrid CNN-LSTM-GRU Model with Data Balancing and XAI Integration. Algorithms.

[22] Hemdan E.E.-D., El-Shafai W., Sayed A. (2023). CR19: A framework for preliminary detection of COVID-19 in cough audio signals using machine learning algorithms for automated medical diagnosis applications. J. Ambient Intell. Humaniz. Comput..

[23] Lundervold A.S., Lundervold A. (2019). An overview of deep learning in medical imaging focusing on MRI. Z. Für Med. Phys..

[24] Emerson F., Divya A. (2018). Performance analysis of brain tumor diagnosis based on soft computing techniques. Int. J. Pure Appl. Math.

[25] Hussain U.N., Khan M.A., Lali I.U., Javed K., Ashraf I., Tariq J., Ali H., Din A. (2020). A unified design of ACO and skewness based brain tumor segmentation and classification from MRI scans. J. Control Eng. Appl. Inform..

